# Evaluation of the upper airway microbiome and immune response with nasal epithelial lining fluid absorption and nasal washes

**DOI:** 10.1038/s41598-020-77289-3

**Published:** 2020-11-26

**Authors:** Meghan H. Shilts, Christian Rosas-Salazar, Christian E. Lynch, Andrey Tovchigrechko, Helen H. Boone, Patty B. Russell, Alexandra S. Connolly, Kaitlin M. Costello, Megan D. McCollum, Annie Mai, Derek A. Wiggins, Seesandra V. Rajagopala, Shibu Yooseph, R. Stokes Peebles, Tina V. Hartert, Suman R. Das

**Affiliations:** 1 Division of Infectious Diseases, Department of Medicine, Vanderbilt University Medical Center, 1161 21st Avenue South, Medical Center North, Suite A2200, Nashville, TN 37232 USA; 2 Department of Pediatrics, Vanderbilt University Medical Center, Nashville, TN USA; 3 Department of Medicine, Allergy, Pulmonary, and Critical Care Medicine, Vanderbilt University Medical Center, Nashville, TN USA; 4 AstraZeneca, Gaithersburg, MD USA; 5 Department of Computer Science, University of Central Florida, Orlando, FL USA; 6 Department of Otolaryngology, Vanderbilt University Medical Center, Nashville, TN USA; 7 Department of Pathology Microbiology and Immunology, Vanderbilt University Medical Center, Nashville, TN USA; 8 Division of Allergy, Pulmonary, and Critical Care Medicine, Department of Medicine, Vanderbilt University Medical Center, 2525 West End Avenue, Suite 450, Nashville, TN 37232 USA

**Keywords:** Clinical microbiology, Metagenomics, Microbiome, Cytokines

## Abstract

Despite being commonly used to collect upper airway epithelial lining fluid, nasal washes are poorly reproducible, not suitable for serial sampling, and limited by a dilution effect. In contrast, nasal filters lack these limitations and are an attractive alternative. To examine whether nasal filters are superior to nasal washes as a sampling method for the characterization of the upper airway microbiome and immune response, we collected paired nasal filters and washes from a group of 40 healthy children and adults. To characterize the upper airway microbiome, we used 16S ribosomal RNA and shotgun metagenomic sequencing. To characterize the immune response, we measured total protein using a BCA assay and 53 immune mediators using multiplex magnetic bead-based assays. We conducted statistical analyses to compare common microbial ecology indices and immune-mediator median fluorescence intensities (MFIs) between sample types. In general, nasal filters were more likely to pass quality control in both children and adults. There were no significant differences in microbiome community richness, α-diversity, or structure between pediatric samples types; however, these were all highly dissimilar between adult sample types. In addition, there were significant differences in the abundance of amplicon sequence variants between sample types in children and adults. In adults, total proteins were significantly higher in nasal filters than nasal washes; consequently, the immune-mediator MFIs were not well detected in nasal washes. Based on better quality control sequencing metrics and higher immunoassay sensitivity, our results suggest that nasal filters are a superior sampling method to characterize the upper airway microbiome and immune response in both children and adults.

## Introduction

The upper airway microbiome and immune response are important determinants of health and studying these is a critical part of ongoing research in the allergy, immunology, and pulmonary fields^1,2^. However, little research has been done to identify the best sampling method for the characterization of the upper airway microbial communities and immune-response signatures.

Despite being the most common technique used for in vivo collection of upper airway epithelial lining fluid^3–5^, nasal washes are occasionally not tolerated well when performed (particularly by children), not suitable for serial sampling, poorly reproducible, and limited by an unknown dilution effect^4–9^. In contrast, nasosorption methods (e.g., nasal filters) lack these limitations and, by directly absorbing the upper airway epithelial lining fluid, have recently emerged as an attractive alternative to nasal washes^4^. We and others have previously shown that nasal filters are well tolerated, easy-to-use, and suitable for multiple research applications^5,7,8,10–13^. However, whether nasosorption methods are superior to nasal washes as a sampling method for studies of the upper airway microbiome and immune response is largely unknown. To address this gap in knowledge, we conducted a cross-sectional study in a group of healthy children and adults.

## Methods

For the whole study, all methods were carried out in accordance with relevant guidelines and regulations.

### Study population and sample collection

For this study, we sampled the upper airway of 40 healthy participants (20 children [mean age (SD) = 3.10 (0.1) years, 60% females] and 20 adults [mean age (SD) = 34.80 (13.02) years, 70% females]) with paired nasal filters and washes. The nasal filter method consisted of introducing ~ 1/3 to 1/2 of a 15 × 25 mm filter paper made of synthetic absorptive matrix (Leukosorb, Pall Life Sciences) into one of the nostrils and placing it laterally against the anterior portion of the inferior nasal turbinate. We then briefly compressed the nasal alae together (in children) or pressed the nasal filter against the lateral wall of the nose with a gloved finger (in adults) to facilitate absorption of the upper airway epithelial lining fluid. The nasal filter was left in the nostril for a minimum of 30 s and up to 2 min. Once removed, it was placed into a sterile container. The nasal wash method consisted of using a bulb syringe to flush 3–5 mL of sterile, non-bacteriostatic, normal saline solution into the contralateral nostril. Both nasal filters and washes were transferred to a freezer and stored at − 80 °C until further processing. The paired samples of all 20 children and 10 of the 20 adults were used for characterization of the upper airway microbiome as described below. In addition, the paired samples of the remaining 10 adults were used for characterization of the upper airway immune response as described below.

One parent of each child and all adults provided informed consent for participation in this study. The Institutional Review Board of Vanderbilt University Medical Center approved this study.

### Bacterial DNA isolation

The PowerSoil DNA Isolation Kit (QIAGEN) was used to extract bacterial DNA from paired samples of all 20 children and 10 of the 20 adults. To this end, we placed nasal filters in 700 µl PowerBead buffer (QIAGEN), vortexed them for 5 min, and removed 600 µl of the supernatant to use with the isolation kit. For nasal washes, 100 µl of the fluid was used. To mechanically lyse bacterial cells, all samples were shaken at 20 Hz in a TissueLyser II system (QIAGEN) for 20 min (for pediatric samples only) or vortexed for 20 min at maximum speed (for adult samples only). One extraction negative control was processed alongside each batch of samples.

### Characterization of the upper airway microbiome with 16S ribosomal RNA sequencing

Following bacterial DNA isolation, the paired samples were processed to prepare sequencing libraries by PCR amplification of the hypervariable V4 region of the bacterial 16S ribosomal RNA (rRNA) gene using universal primers. Both negative and positive controls (with known taxonomic composition) were amplified alongside all samples. We then sequenced the pooled libraries on an Illumina MiSeq platform with either 2 × 250 (in children) or 2 × 300 (in adults) base pair reads.

The 16S rRNA datasets were processed in R^14^ using the *dada2* pipeline by following its standard operating procedure (available at: https://benjjneb.github.io/dada2/tutorial.html)^15^. To this end, sequences were grouped into amplicon sequence variants (ASVs) and taxonomy was assigned using the Ribosomal Database Project reference dataset^16^. Sequences were subsequently processed through the R package *decontam* to remove any suspected contaminants that were found in the negative control samples^17^. Further details on library construction and data processing are available in the E-Methods section of the Online Repository.

### Characterization of the upper airway microbiome with shotgun metagenomic sequencing

To complement the 16S rRNA sequencing, we also performed shotgun metagenomic sequencing in paired samples from a subset of children (n = 8). First, eukaryotic DNA was depleted using the NEBNext Microbiome Enrichment Kit (New England Biolabs). Next, we constructed dual-indexed sequencing libraries with the Nextera XT DNA Library Prep Kit (Illumina). Equimolar amounts of each library were then pooled and sequencing was performed on 5% of an Illumina NovaSeq6000 platform (S4 flow cells run) with 2 × 150 base pair reads. For the initial processing of the shotgun whole metagenomic sequencing dataset, we used FastQC to assess the sequence quality^18^. The adapter trimming and removal of low-quality data were performed with Trimmomatic^19^. Following this, we removed human DNA by aligning sequences to the Genome Research Consortium Human Build 38 reference assembly with the Burrows*-*Wheeler Aligner^20,21^. Last, we assigned taxonomy using GOTTCHA^22^.

### Characterization of the upper airway immune response

The upper airway immune signatures were profiled in paired samples from 10 adults using 2 multiplex magnetic bead-based assays (Milliplex MAP Human Cytokine/Chemokine Magnetic Bead Panel II Premixed 23 Plex Kit [MilliporeSigma] and Cytokine 30-Plex Human Panel [Thermo Fisher Scientific]), as previously described^12,23^. These panels measure a total of 53 immune mediators (i.e., cytokines, chemokines, and growth factors) that have been associated with clinical outcomes in allergy, immunology, and pulmonary research (Table E1). Prior to running the assay, filters were eluted into 300 µl of 0.9% NaCl solution. For each sample type, 20 µl was run on each multiplex plate. The assays were all conducted in duplicate on a Luminex MAGPIX platform. One blank well was used as a negative control.

To avoid potential bias in immune mediator readings due to a dilution effect between washes and filters, we measured total protein in adult nasal wash and filter samples with the Pierce BCA Protein Assay Kit (ThermoFisher). For all samples, 25 µl of each sample was used for the assay. This volume allowed detection down to 20 µg/ml. Saline was used as the negative control. Samples were run in triplicate and protein concentrations were averaged.

### Statistical analyses

For the 16S rRNA dataset, the statistical analyses were conducted in R^14^, mostly using the open-source package MGSAT^24^, as previously described^10,11,25,26^. The MGSAT pipeline wraps several R packages to compare common microbial ecology indices of community richness (e.g., Chao1, Jackknife, and Bootstrap estimators), α-diversity (e.g., Hill numbers N1 and N2, which are equivalent to the exponentiated Shannon and inverted Simpson indices, respectively), and structure (e.g., Bray–Curtis dissimilarities) between groups. For the assessment of community structure, MGSAT uses the *vegan* package to test for differences in Bray–Curtis dissimilarities and homogeneity of variances between groups with the PermANOVA (*Adonis* function) and *betadisper* tests, respectively^27,28^. For the PermANOVA test, strata was set as subject study numbers. To compare the abundance of taxa between groups, we used the *DESeq2* test^29^, as implemented in MGSAT, which uses a Wald test with the Benjamini–Hochberg correction to control for multiple comparisons^30^. We included subject study number as a covariate in all *DESeq2* models. Because the number of paired samples with shotgun metagenomic sequencing data was small, we did not perform any statistical comparisons between groups and only compared these using descriptive statistics.

The statistical analyses of the immune mediator dataset were also conducted in R^14^. The Luminex xMAP data was processed using a method that uses median fluorescence intensities (MFIs) of individual beads instead of the usual standard curve-based data-processing method to increase the sensitivity and accuracy of high-throughput immunoassays^23^. The median fluorescence intensity (MFI) of each of the 53 analytes was calculated after subtracting out the background MFI and this was used for all statistical analyses. Because the MFIs were not normally distributed, these were log10 transformed. Prior to this, all negative MFIs were set to 0, as a value < 0 indicates that the MFI for that particular analyte was lower than the background MFI (i.e., that the analyte was not truly detected in that sample). The MFIs were then transformed using the equation log10(x + 1) to allow analyte readings of 0 to remain 0. The comparisons of MFIs between paired samples were performed using a Wilcoxon signed-rank test with the Benjamini–Hochberg correction to control for multiple comparisons. For total protein concentration measurements, absorbance were obtained with the BCA (bicinchoninic acid) assay. Concentrations were calculated from the standards. The student’s paired *t* test was used to test for significance of protein concentrations between paired filters and washes.

For data visualization, we used grouped or stacked bar graphs, box and whisker plots, heatmaps, and ordination plots based on different microbial ecology indices or immune mediators MFIs, as appropriate. Figures were generated with the R packages *ggplot2*^31^, *vegan*^32^, or *ComplexHeatmap*^33^, as appropriate. Heatmaps were generated with *ComplexHeatmap*^33^; the Pearson correlation was used to calculate dissimilarities. The number of cluster splits was determined by partitioning around medoids (method pamk in R package *fpc*^34^). Minor aesthetic edits to the figure were performed in Inkscape version 1.0. Statistical significance was defined as *p*- or *q*-values < 0.05. Due to the differences in sample processing steps between children and adults, all results are presented separately for each of these age groups. Further details on the study methods are available in the E-Methods section of the Online Repository.

## Results

### Comparison of the upper airway microbiome between pediatric nasal filters and washes using 16S ribosomal RNA sequencing

Following all the 16S rRNA data processing steps, the median (interquartile range [IQR]) retained sequence count per sample among all pediatric samples was 15,858 (4777–21,149). The median (IQR) retained sequence count per sample was 20,369 (12,050–21,695) among nasal filters and 8,927 (431–17,853) among nasal washes (Wilcoxon signed-rank test *p* = 0.04). Because samples with few reads are likely to represent environmental contamination, those with < 1000 reads (n = 7) were discarded. Of the 20 paired samples from children with 16S rRNA data, 19 nasal filters (95%) and 14 nasal washes (70%) had > 1000 reads (Fisher’s exact *p*-value = 0.09). Twenty-six of these remaining samples were paired and were thus included in further analyses.

**Figure 1:**
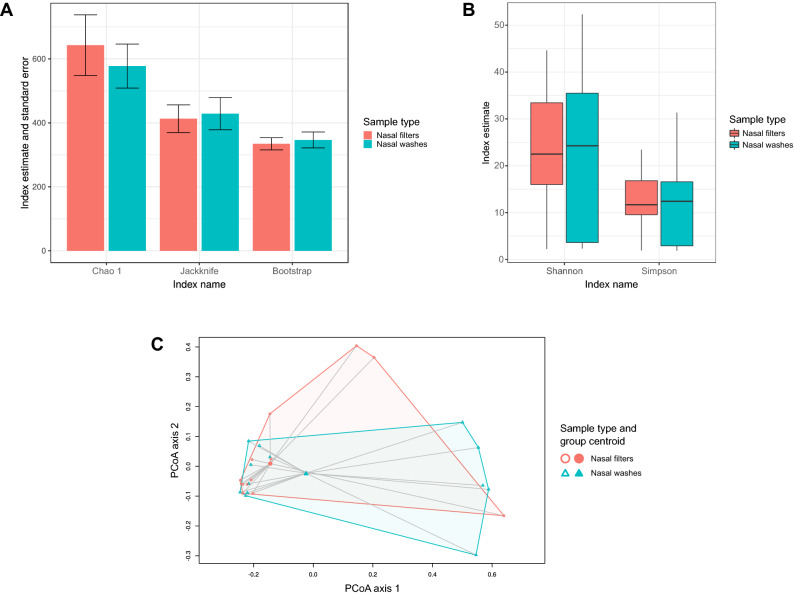
Common microbial ecology indices of the upper airway microbiome in children with paired samples based on 16S ribosomal RNA sequencing and according to sample type. (**A**) Grouped bar graphs of selected indices showing no differences in community richness between nasal filters and nasal washes at the amplicon sequence variant unit (ASV) level. (**B**) Box-and-whisker plots of selected indices showing no differences in community α-diversity between nasal filters than in nasal washes at the ASV level. (**C**) Principal coordinates analysis (PCoA) plot of Bray–Curtis dissimilarities showing no distinct clustering by sample type at the ASV level. The lines connect samples with their group centroids. (**A**,**B**) were generated with the R^14^ package *ggplot2* version 3.0.0 (https://cran.r-project.org/web/packages/ggplot2/index.html);^31^ (**C**) was generated in *vegan* version 2.5-2 (https://cran.r-project.org/web/packages/vegan/index.html)^32^ and minor aesthetic edits were performed with Inkscape version 1.0.

There were no differences in community richness (Fig. 1A) or α-diversity (Fig. 1B) between sample types at the ASV level in children (*p* > 0.05 for all comparisons). The community structure at the ASV level was also similar between nasal filters and washes (PermANOVA test *p* = 0.3 and *betadisper* test *p* = 0.2) (Fig. 1C). Overall, 174 ASVs were found in both filters and washes, while 161 ASVs were found only in nasal washes, and 159 ASVs were found only in nasal washes (Figure S1A).

**Figure 2:**
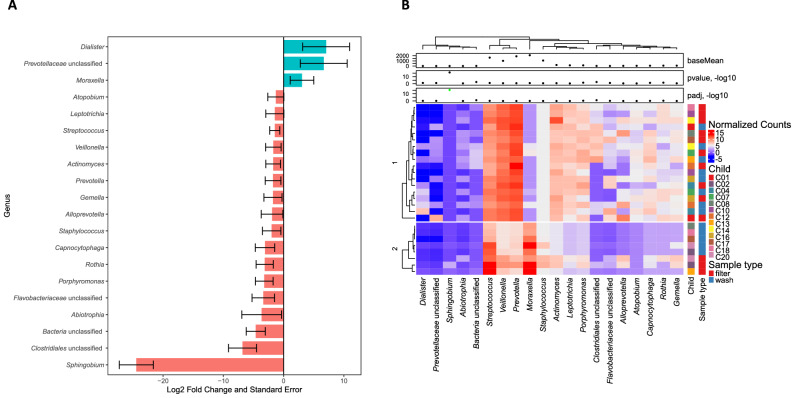
(**A**) Log_2_ fold change and log_2_ fold change standard error of nasal bacterial genera according to sample type in children as calculated with the paired *DESeq2* analysis. The log_2_ fold change of the 20 most differentially abundant bacterial genera are shown. A log_2_ fold change of > 0 (pink bars) indicates that abundance was detected to be higher in the nasal filters as compared to washes, while a log_2_ fold change < 0 (blue bars) indicates that abundance was detected to be higher in nasal washes compared to nasal filters. After the Benjamini–Hochberg correction for multiple comparisons, only *Sphingobium* remained significantly differentially abundant between sample types. This figure was generated with the R^14^ package *ggplot2* version 3.0.0 (https://cran.r-project.org/web/packages/ggplot2/index.html)^31^. (**B**) Hierarchically clustered heatmap of upper airway genera abundance in children with paired samples based on 16S ribosomal RNA sequencing and according to sample type. Only the top 20 genera with the lowest q-values are shown. The abundance of each genus is shown as its base mean, which represents the mean of counts of that particular genus in all samples after normalizing these by library size, and as regularized counts, which are calculated for each sample by transforming the normalized counts to the log2 scale. The heatmap cell colors represent the regularized counts as shown in the color scale. The log2-fold change in the abundance of each genus is also shown. P-values and adjusted p-values are shown; a green dot indicates the adjusted p-value was < 0.05. One genus, *Sphingobium*, was differentially abundant between nasal filters and washes with the *DESeq2* test after controlling for multiple comparisons with the Benjamini–Hochberg correction (*q*-value < 0.05). This figure was generated with the R^14^ package *ComplexHeatmap* version 1.18.1 (https://www.bioconductor.org/packages/release/bioc/html/ComplexHeatmap.html)^33^.

There were a total of 81 and 79 genera identified in pediatric nasal filters and washes, respectively. The most abundant genera in nasal filters were *Prevotella* (29%), *Veillonella* (14%), *Streptococcus* (10%), *Moraxella* (10%), *Haemophilus* (5%), *Neisseria* (4%), and *Actinomyces* (3%), whereas the most abundant genera in nasal washes were *Moraxella* (21%), *Prevotella* (20%), *Veillonella* (12%), *Haemophilus* (11%), *Streptococcus* (6%), *Dolosigranulum* (4%), *Neisseria* (3%), and *Corynebacterium* (3%); all other genera were present at < 3% mean relative abundance. At the genus level, only *Sphingobium* was differentially abundant between paired samples (log2 fold change = − 24.4456, *q*-value = 3.93E−16) with the *DESeq2* test (Fig. 2 and Table E2 in the Online Repository). Using the *DESeq2* test at the ASV level, 22 ASVs were differentially abundant between paired sample types (Table E3 in the Online Repository).

### Comparison of the upper airway microbiome between pediatric nasal filters and washes using shotgun metagenomic sequencing

Following all the shotgun metagenomic sequencing data processing steps, the median (IQR) retained sequence count per sample among all pediatric samples was 738,821 (538,420–1,855,491). The median (IQR) retained sequence count per sample was 621,994 (296,015–700,034) among nasal filters and 1,616,436 (1,244,683–3,317,855) among nasal washes. Of the 8 paired samples undergoing shotgun metagenomic sequencing in children, 2 samples failed to sequence, leaving 7 nasal filters (88%) and 7 nasal washes (88%) that sequenced successfully. Twelve of these samples were paired and were thus included in further analyses.

**Figure 3:**
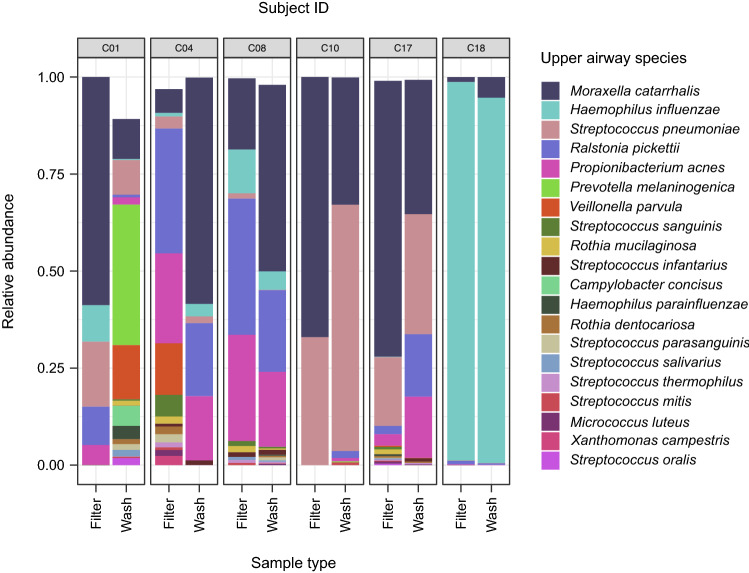
Stacked bar graphs of the abundance of upper airway species in children with paired samples based on shotgun metagenomic sequencing and according to sample type. Only the top 20 most abundant species are shown. The species abundances are shown as relative abundances, which are calculated as simple proportions by dividing the observed sequence count of a species in a sample by the total count of sequences in that particular sample. This figure was generated with the R^14^ package *ggplot2* version 3.0.0 (https://cran.r-project.org/web/packages/ggplot2/index.html).

There were a total of 36 and 60 species identified in pediatric nasal filters and washes, respectively. Rarefaction curves level off for most of the samples, suggesting that sequencing was generally performed to an adequate depth to identify the bacteria in the samples (Figure S2). The most abundant species in nasal filters were *Moraxella catarrhalis* (37%), *Haemophilus influenzae* (21%), *Streptococcus pneumoniae* (14%)*, Ralstonia pickettii* (13%) and *Propionibacterium acnes* (9%); whereas the most abundant species in nasal washes were *Moraxella catarrhalis* (35%), *Streptococcus pneumoniae* (19%) *Haemophilus influenzae* (16%), *Ralstonia pickettii* (8%), and *Propionibacterium acnes* (8%); all other species were present at < 6% mean relative abundance. Even though the predominant species detected by nasal filters and washes were usually the same, their relative abundances were not always consistent among sample types (Fig. 3).

### Comparison of the upper airway microbiome between adult nasal filters and washes using 16S ribosomal RNA sequencing

Following all the 16S rRNA data processing steps, the median (IQR) retained sequence count per sample among all adult samples was 11,823 (6773–19,812). The median (IQR) retained sequence count per sample was 10,547 (6123–12,238) among nasal filters and 18,288 (7891–24,337) among nasal washes (Wilcoxon signed-rank test *p* = 0.1). Of the 10 paired samples undergoing 16S rRNA sequencing in adults, all 10 nasal filters and 10 washes had > 1000 reads and, thus, these were all included in further analyses.

**Figure 4:**
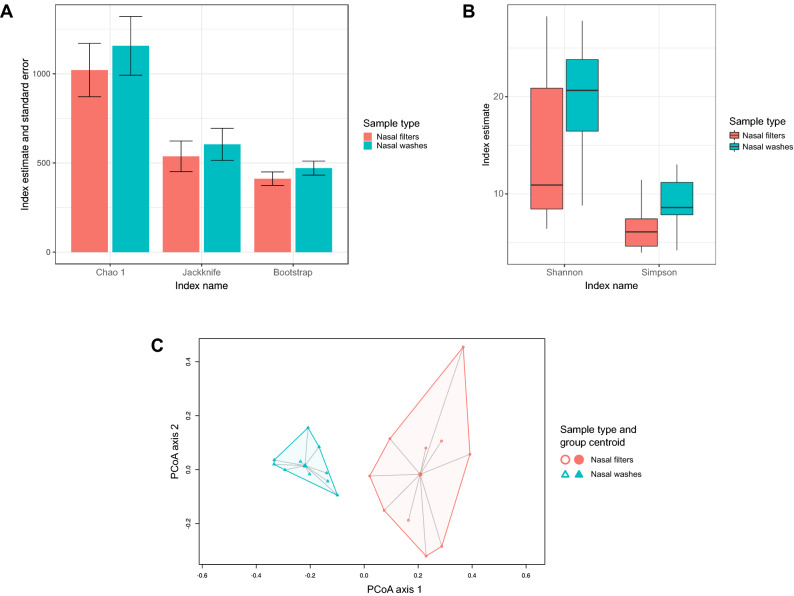
Common microbial ecology indices of the upper airway microbiome in adults with paired samples based on 16S ribosomal RNA sequencing and according to sample type. (**A**) Grouped bar graphs of selected indices showing higher community richness in nasal filters than in nasal washes at the amplicon sequence variant (ASV) level. (**B**) Box-and-whisker plots of selected indices showing higher community α-diversity in nasal filters than in nasal washes at the ASV level. (**C**) Principal coordinates analysis (PCoA) plot of Bray–Curtis dissimilarities showing distinct clustering by sample type at the ASV level. The lines connect samples with their group centroids. (**A**,**B**) were generated with the R^14^ package *ggplot2* version 3.0.0 (https://cran.r-project.org/web/packages/ggplot2/index.html)^31^; (**C**) was generated in *vegan* version 2.5–2 (https://cran.r-project.org/web/packages/vegan/index.html)^32^ and minor aesthetic edits were performed with Inkscape.

There were substantial differences in community richness (Fig. 4A) and α-diversity (Fig. 4B) between samples types at the ASV level in adults (*p* < 0.05 for all comparisons). The community structure at the ASV level was also highly dissimilar between nasal filters and washes (PermANOVA test *p* = 0.002 and *betadisper* test *p* = 0.003) (Fig. 4C). Overall, 104 ASVs were found in both filters and washes, while 290 ASVs were found only in nasal washes, and 226 ASVs were found only in nasal washes (Figure S1B).

**Figure 5:**
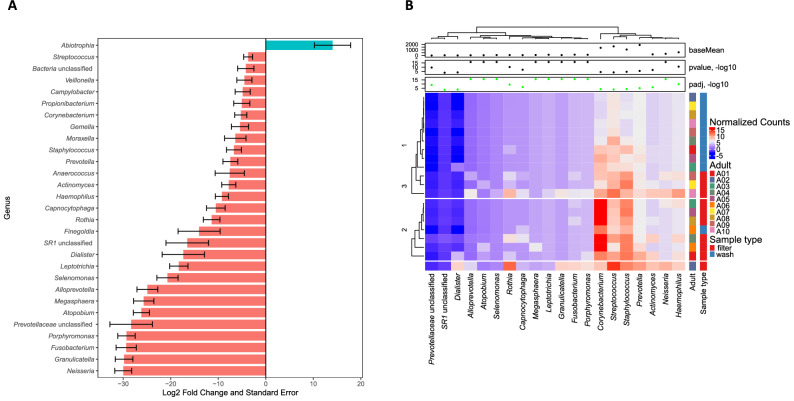
(**A**) Log_2_ fold change and log_2_ fold change standard error of nasal bacterial genera according to sample type in adults as calculated with the paired *DESeq2* analysis. The log_2_ fold changes of the 29 differentially abundant bacterial genera are shown. A log_2_ fold change of > 0 (pink bars) indicates that abundance was detected to be higher in the nasal filters as compared to washes, while a log_2_ fold change < 0 (blue bars) indicates that abundance was detected to be higher in nasal washes compared to nasal filters. After the Benjamini–Hochberg correction for multiple comparisons, 29 genera were differentially abundant between nasal filters and washes. This figure was generated with the R^14^ package *ggplot2* version 3.0.0 (https://cran.r-project.org/web/packages/ggplot2/index.html)^31^. (**B**) Hierarchically clustered heatmap of upper airway genera abundance in adults with paired samples based on 16S ribosomal sequencing and according to sample type. Twenty-nine genera were differentially abundant between nasal filters and washes with the *DESeq2* test after controlling for multiple comparisons with the Benjamini–Hochberg correction (*q*-value < 0.05 for all comparisons). Only the 20 top genera with the lowest *q*-values are shown. The abundance of each genus is shown as its base mean, which represents the mean of counts of that particular genus in all samples after normalizing these by library size, and as regularized counts, which are calculated for each sample by transforming the normalized counts to the log2 scale. The heatmap cell colors represent the regularized counts as shown in the color scale. The log2-fold change in the abundance of each genus is also shown. P-values and adjusted p-values are shown; a green dot indicates the adjusted p-value was < 0.05. This figure was generated with the R^14^ package *ComplexHeatmap* version 1.18.1 (https://www.bioconductor.org/packages/release/bioc/html/ComplexHeatmap.html)^33^.

There were a total of 141 and 188 genera identified in adult nasal filters and washes, respectively. The most abundant genera in nasal filters were *Corynebacterium* (20%), *Staphylococcus* (12%), and *Streptococcus* (9%); whereas the most abundant genera in nasal washes were *Cloacibacterium* (28%), *Acidovorax* (13%); *Comamonas* (9%), *Dechloromonas* (7%), *Aquabacterium* (6%), and *Corynebacterium* (5%); all other genera were present at < 5% mean relative abundance. The abundances of 29 genera (Fig. 5 and Table E2 in the Online Repository) and 69 ASVs (Table E3 in the Online Repository) were different between paired samples (*q* < 0.05 for all comparisons).

### Comparison of the upper airway immune response between adult nasal filters and washes

**Figure 6:**
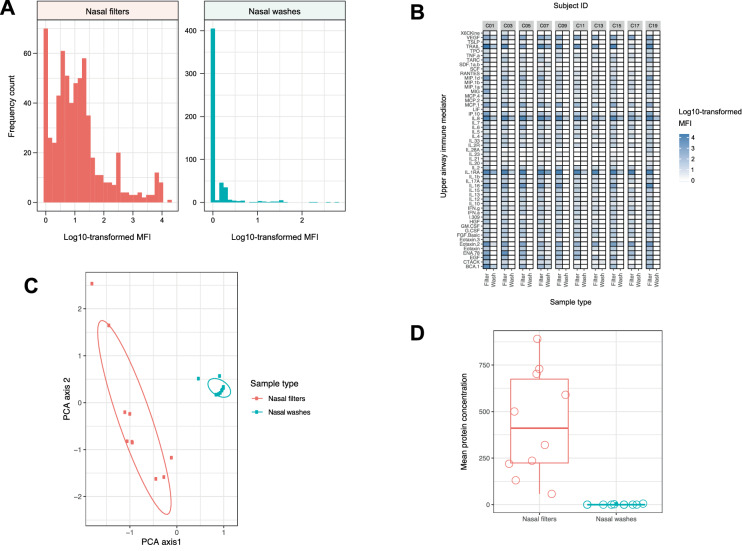
Upper airway immune-response signatures in adults with paired samples based on the measurement of 53 immune mediators and according to sample type. (**A**) Histograms of log10-transformed median fluorescence intensities (MFIs) showing that immune mediators were detected more frequently in nasal filters than in nasal washes. (**B**) Unclustered heatmap showing that the log10-transformed MFIs of immune mediators were generally higher in nasal filters than in nasal washes for each pair of samples. The heatmap cell colors represent log10-transformed MFIs as shown in the color scale. (**C**) Principal component analysis (PCA) plot of immune-response signatures showing distinct clusters by sample type. (**D**) Box and whisker plot showing mean protein concentration as detected with the BCA assay. Open circles represent individual subject sample readings. Protein concentration was significantly higher in nasal filters compared to the nasal washes. All panels of this figure were generated with the R^14^ package *ggplot2* version 3.0.0 (https://cran.r-project.org/web/packages/ggplot2/index.html)^31^.

In the 10 adult paired samples with immune mediator data, the 53 analytes tested were detected more frequently in nasal filters (482/530 [91%]) than in nasal washes (125/530 [24%]) (Fisher’s exact test *p* < 0.001) (Fig. 6A). In nasal washes, only IL-1RA and IL-8 were consistently detected, whereas nearly all immune mediators were detected in nasal filters (mostly with medium to high MFIs). Except for IL-21, IL-28A, LIF, SDF-1A + β, TPO, and 6Ckine, the MFIs of the other 47 immune mediators tested were all higher in nasal filters than in nasal washes (*q* < 0.05 for all comparisons) (Fig. 6B and Table E4 in the Online Repository). The immune-response signatures were also highly dissimilar between sampling methods, distinctly clustering by sample type (Fig. 6C).

We examined the total protein in adult nasal washes and filters with a BCA assay (Fig. 6D). Protein concentrations detected in washes were similar to that of saline blank controls and were calculated to be 0 for 8/10 washes (median [IQR] 0 [0–0] µg/ml). In contrast, the median (IQR) concentration reading in filters was 410.61 (223.6–674.56) µg/ml. Total protein concentration in washes was significantly lower than that of filters (student’s paired *t* test *p* = 0.0009).

## Conclusions

There is a need for novel methods of in vivo collection of upper airway epithelial lining fluid to improve the rigor and reproducibility of research in the allergy, immunology, and pulmonary fields^4^. In our study, we found that, for the characterization of the upper airway microbial communities and immune-response signatures, (1) nasal filters are overall superior to nasal washes as a sampling method, and (2) the upper airway sampling method can have a large impact in the study results, particularly in adults. Our results measuring both total protein concentration and levels of individual immune mediators suggest that, in contrast to nasal filters, proteins are not well-captured in nasal washes in adults. Previous research has demonstrated that, although less frequently used, nasal filters are easier to obtain and less prone to bias than nasal washes in both children and adults^5,7,8,10–13,35,36^, and our study adds to the literature supporting the superiority of nasal filters as an upper airway sampling method by showing they are more sensitive for both microbiome and immune assays.

In our prior studies of the upper airway microbiome, we have shown that nasal filters can be easily used in children younger than 6 months of age^10,11^. Nasal filters are well-tolerated and are particularly suitable for neonates (who are preferentially obligate nasal breathers^37^) and preschool-aged children (who tend to be poorly cooperative). It is also our experience that, although usually rare, the potential risks associated to nasal washes (e.g., upper airway trauma, pain, discomfort, and pulmonary aspiration) can be enough to discourage parents for enrolling their children in certain studies. Thus, the use of nasal filters could increase participation in pediatric research. Unlike other studies that have left nasal filters in the nostrils for up to 10 min^5,7,35,36,38^, we were able to collect upper epithelial lining fluid leaving them for a minimum of 30 s. This shorter time improves tolerability and further facilitates its use in the pediatric population.

In spite of the importance of selecting the best sampling method as part of the design of studies of the upper airway microbial communities, no prior study had compared nasal filters to nasal washes to sample the upper airway microbiome, and this is the first study to show that a sufficient quantity of high-quality microbial DNA can be obtained from the nasal filters to perform both 16S rRNA and shotgun metagenomic sequencing. Furthermore, other studies comparing these sampling methods for the evaluation of upper airway immune-response signatures have measured fewer analytes^5,7,8^. In one study (n = 16) assessing 5 immune mediators, the percent of samples with levels of IL-8, IL-1β, TNF-α, eosinophilic cationic protein, and tryptase above the assay’s lower limit of detection were consistently higher among nasal filters (100%, 100%, 67%, 86%, and 33%, respectively) than among nasal washes (93%, 94%, 57%, 71% and 8%, respectively)^7^. Likewise, the levels of IL-8, IL-1β, IP-10, and neutrophil elastase (but not IL-6) were higher in nasal filters than in nasal washes in one other study (n = 10), in many cases by several orders of magnitude^8^. In a more recent study (n = 6) assessing 30 immune mediators with one of the multiplex magnetic bead-based assays we used, all analytes were detected in nasal filters but not in nasal washes (with the immune mediator levels being ~ 4.7 times higher in nasal filters)^5^. Other studies have shown that nasal filters can be useful for the assessment of respiratory viruses, certain molecules (e.g., lactoferrin), and bacterial RNA expression^7,8^. In contrast to nasal washes—which should only be used once a day^6^—nasal filters can also be used multiple times a day, making them ideal for serial sampling and longitudinal studies of the upper airway microbiome and immune response^7,12,39^.

The comprehensive characterization of the upper airway microbiome using different next-generation sequencing techniques, the use of multiple statistical analyses, the measurement of a large number of immune mediators, and the inclusion of both children and adults are all important strengths of our study. We should also acknowledge several limitations. First, our study’s sample size was small, so it is possible that we were underpowered to detect some differences between sample types. However, we were still able to show large differences between the sampling methods (particularly in adults). Of note, because some of the laboratory methods used to characterize the microbiome in adult pediatric and adult samples differed, we could not directly compare these age groups and we have presented results separately for each of them. Second, nasal washes likely retrieve epithelial lining fluid from more distal regions of the upper airway than nasal filters (e.g., the nasopharynx), which could explain some of the differences we found. This may also explain why the differences in sampling methods were more obvious in adults, who have upper airways that are larger than those of children. Third, we did not compare nasal filters to other commonly used techniques that could be equally effective as sampling methods of the upper airway (e.g., nasal swabs). Other nasosorption methods (e.g., cotton strips, polyurethane foam, and cellulose sponges) have also been used in adult studies of the upper airway immune response and could potentially be used to assess the microbiome, although this has not been studied and these techniques may be harder to perform in children^3,40,41^. Fourth, our study only focused on sampling methods of the upper airway. In addition to being the initial site of respiratory bacterial colonization, the nostrils are the portal of entry for aeroallergens, air pollutants, and respiratory viruses, and thus are of critical importance in the pathogenesis of allergic, immunologic, and pulmonary diseases in both children and adults. Furthermore, it is impractical to obtain lower airway samples from healthy children, so most pediatric studies in these fields have traditionally sampled the upper airway. Of note, nasosorption methods to sample the lower airways have also been recently developed^35^. Fifth, we did not sequence unused filters or the saline solution that passed through the bulb syringe as part of this study, so there could be some degree of residual contamination that was not taken into account. Last, due to the concern that one sampling method may alter the results of the subsequent sampling method, we did not use both sampling methods in the same participant’s nostril. However, other studies have shown that there is minimal intra-subject variability in regard to upper airway microbial communities and immune-response signatures between nostrils^8,42^.

In summary, we found that (1) nasal filters are a superior sampling method to characterize upper airway microbial communities and immune-response signatures when compared to nasal washes in both children and adults, (2) nasal filters can be used as a sampling method for studies examining the upper airway microbiome using both 16S rRNA and shotgun metagenomic sequencing. Based on this, future studies examining the upper airway microbiome and immune response should consider using nasal filters as their preferred sampling method.

## Supplementary information

Supplementary Figure S1.

Supplementary Figure S2.

Supplementary Information.

## Abbreviations

ASV: Amplicon sequence variant
IQR: Interquartile range
MFI: Median fluorescence intensity
rRNA: Ribosomal ribonucleic acid
BCA: Bicinchoninic acid
