# Supplementary figures and images for: Evaluation of the upper airway microbiome and immune response with nasal epithelial lining fluid absorption and nasal washes
Source: Sci Rep. 2020 Nov 26;10:20618. doi: 10.1038/s41598-020-77289-3 (PMC7692476; doi:10.1038/s41598-020-77289-3)

**A**

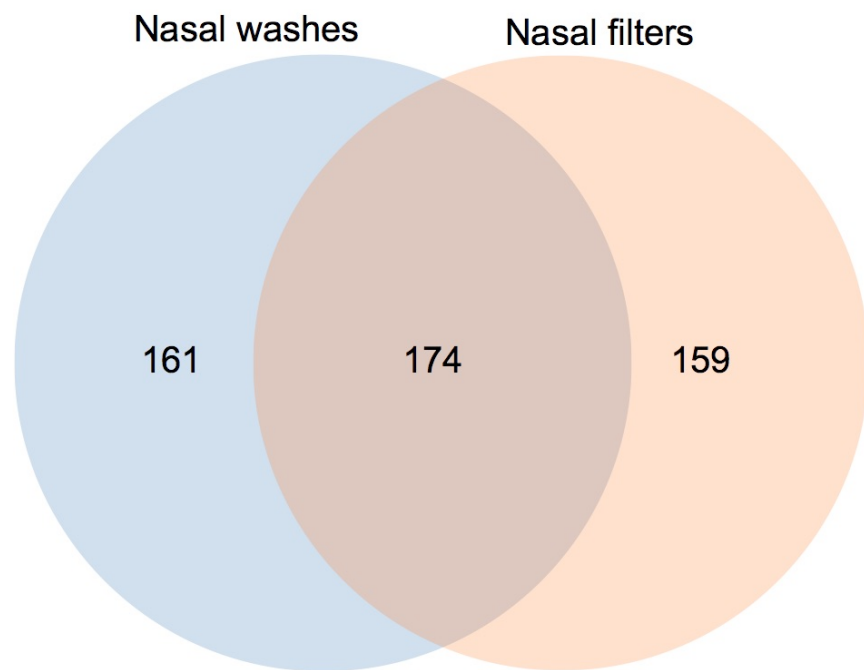

**B**

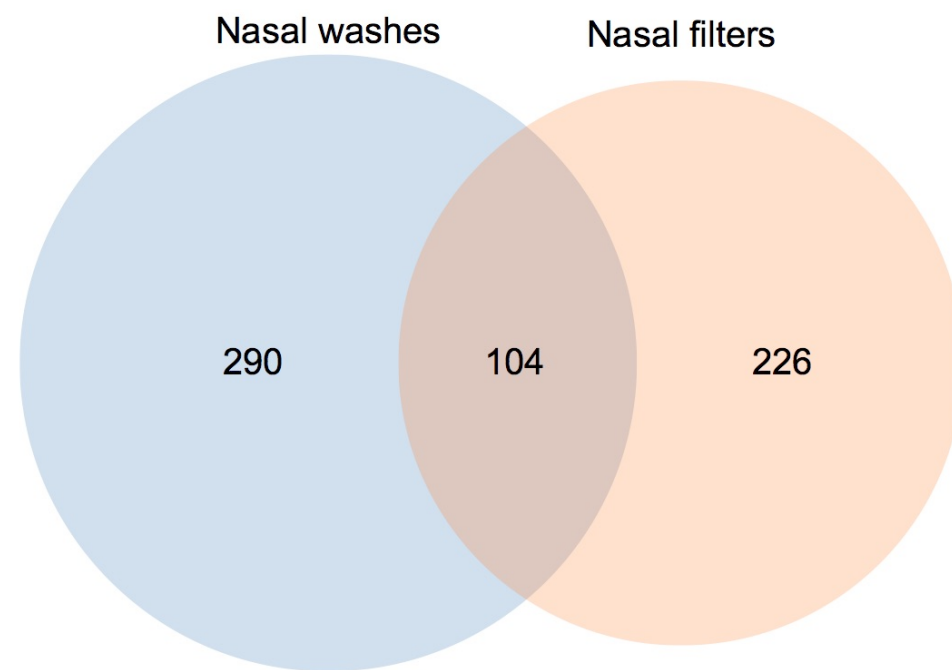

Supplement: Supplementary file 1 — Supplementary Figure S1. [file 41598_2020_77289_MOESM1_ESM.pdf]
