# Supplementary material for: Evaluation of the upper airway microbiome and immune response with nasal epithelial lining fluid absorption and nasal washes
Source: Sci Rep. 2020 Nov 26;10:20618. doi: 10.1038/s41598-020-77289-3 (PMC7692476; doi:10.1038/s41598-020-77289-3)
Supplement: Supplementary file 2 — Supplementary Figure S2. [file 41598_2020_77289_MOESM2_ESM.pdf]

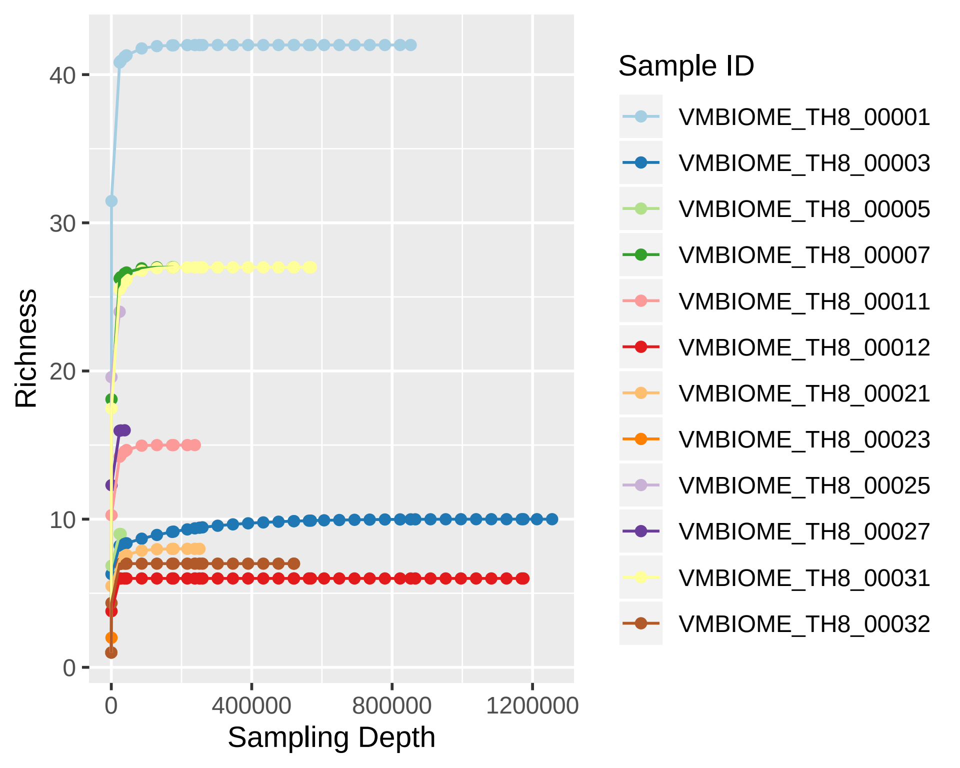

Richness
40
30
20
10
0
0
400000
800000
1200000
Sampling Depth
Sample ID
VMBIOME_TH8_00001
VMBIOME_TH8_00003
VMBIOME_TH8_00005
VMBIOME_TH8_00007
VMBIOME_TH8_00011
VMBIOME_TH8_00012
VMBIOME_TH8_00021
VMBIOME_TH8_00023
VMBIOME_TH8_00025
VMBIOME_TH8_00027
VMBIOME_TH8_00031
VMBIOME_TH8_00032
