# Supplementary material for: Evaluation of the upper airway microbiome and immune response with nasal epithelial lining fluid absorption and nasal washes
Source: Sci Rep. 2020 Nov 26;10:20618. doi: 10.1038/s41598-020-77289-3 (PMC7692476; doi:10.1038/s41598-020-77289-3)
Supplement: Supplementary file 3 — Supplementary Information. [file 41598_2020_77289_MOESM3_ESM.docx]

**Evaluation of the Upper Airway Microbiome and Immune Response with Nasal Epithelial Lining Fluid Absorption and Nasal Washes**

**Online Repository**

Meghan H. Shilts, MHS, MS,^1*^ Christian Rosas-Salazar, MD, MPH,^2*^ Christian E. Lynch, MPH,^3^ Andrey Tovchigrechko, PhD,^4^ Helen H. Boone, BS,^1^ Patty B. Russell, RN,^2^ Alexandra S. Connolly, BS,^2^ Kaitlin M. Costello, BA,^2^ Megan D. McCollum, MS,^2^ Annie Mai, BS,^1^ Derek A Wiggins, MS,^3^ Seesandra V. Rajagopala, PhD,^1^ Shibu Yooseph, PhD,^5^ R. Stokes Peebles, MD,^2^ Tina V. Hartert, MD, MPH,^2#^ and Suman R. Das, PhD^1#^

^*^These authors contributed equally to this study

^#^Corresponding authors

**E-Methods** Page 2

**Table E1** Page 8

**Table E2** Page 9

**Table E3** Page 11

**Table E4** Page 13

**Supplementary Figure Legends** Page 15

**E-References** Page 16

**E-Methods**

***DNA extraction***

All samples were extracted with the Qiagen PowerSoil kit. We attempted to keep the extraction method as similar as possible between the different sample types (i.e., filters and washes). The nasal washes, as a liquid, could immediately be pipetted into 700 µl of the PowerBead buffer, as recommended per the manufacturer’s protocol, and the rest of the extraction protocol could be followed. The nasal filters, as a solid, could not be directly pipetted into the PowerBead buffer. Instead, we added the nasal filter to a tube with 700 µl PowerBead buffer, and vortexed the tube to encourage the bacteria and bacterial DNA to elute into the PowerBead buffer. As much of the PowerBead buffer as could be removed (~600 µl) was then pipetted into the columns, and the protocol was continued as per the manufacturer’s protocol.

***Library preparation and 16R ribosomal RNA sequencing of pediatrics samples***

For pediatric paired samples, sequencing libraries were generated with a one-step amplification process, over 30 total cycles. Dual-indexed universal primers appended with Illumina-compatible adapters were used to amplify the hypervariable V4 region of the bacterial 16S rRNA gene, with PCR parameters as previously described.^1^ Briefly, the hypervariable V4 region of the bacterial 16S ribosomal RNA (rRNA) gene was amplified with primers v4.SA501 5’- AATGATACGGCGACCACCGAGATCTACACNNNNNNNNTATGGTAATTGTGTGCCAGCMGCCGCGGTAA-3’ and v4.SA701 5’- CAAGCAGAAGACGGCATACGAGATNNNNNNNNAGTCAGTCAGCCGGACTACHVGGGTWTCTAAT-3’; in each primer, the 8 sequential Ns represent the index sequence. An initial denaturing step at 95°C was performed for 2 min. This was followed by 30 cycles of 95°C for 20 seconds, 55°C for 15 seconds, and 72°C for 5 minutes, and a final extension at 72°C for 10 minutes.

Each amplified sample was run on a 1% agarose gel to confirm reaction success. All of the negative controls failed to show a visible band at this stage. Amplicons were cleaned and normalized with the SequalPrep Normalization Kit (Thermo Fisher Scientific). Following normalization to 1-2 ng/µl, 10 µl of each sample was combined to create the sequencing pool. Normalized amplicons were pooled and cleaned with 1X AMPure XP (Beckman Coulter) beads. The pool was then sequenced on an Illumina MiSeq platform with 2x250 base pair reads.

The extraction control and a PCR negative control, along with one sample with known taxonomic composition (ZYMOBiomics Microbial Community Standard [Zymo Research]) were amplified and sequenced concurrently with the samples.

***Library preparation and 16R ribosomal RNA sequencing of adult samples***

For adult paired samples, sequencing libraries were generated with a two-step amplification process. During the first round of PCR, the hypervariable V4 region of the bacterial 16S ribosomal RNA (rRNA) gene was amplified with primers 515F 5’-GTGCCAGCHGCYGCGGT-3’ and 806R 5’-GGACTACNNGGGTWTCTAAT-3’, with an initial denaturing step at 95°C for 3 min. This was followed by 10 cycles of 95°C for 30 seconds, 50°C for 30 seconds, and 72°C for 1 second, and a final extension at 72°C for 5 minutes. During the second round of PCR, 30 cycles with the same cycling condition as before were performed to add Illumina adaptors, standard Illumina sequence primer region, a 12 base pair barcode attached to the reverse primer, and random nucleotides to increase sequence diversity.

Each amplified sample was run on a 1.2% agarose gel to confirm reaction success. All of the adult nasal samples showed a visible band at this stage, while all negative controls did not. Amplicons were cleaned and normalized with the SequalPrep Normalization Kit (Thermo Fisher Scientific). Following normalization to 1-2 ng/µl, 10 µl of each sample was combined to create the sequencing pool. Normalized amplicons were pooled and cleaned with 1X AMPure XP (Beckman Coulter) beads. The pool was run on a 1.5% agarose gel and the target size band was extracted and cleaned with the NucleoSpin Gel and PCR Clean-up kit (Macherey-Nagel). The pool was then sequenced on an Illumina MiSeq platform with 2x300 base pair reads.

The extraction control and a PCR negative control, along with 4 controls with known taxonomic composition (provided by the NIH/NIAID Biological and Emerging Infections [BEI] Program)^2^ were amplified and sequenced concurrently with the samples. The BEI controls included: 1) Genomic DNA from Microbial Mock Community B (Staggered, Low Concentration), v5.2L, for 16S rRNA Gene Sequencing, HM- 783D, and 2) Genomic DNA from Microbial Mock Community B (Even, Low Concentration), v5.1L, for 16S rRNA Gene Sequencing, HM-782D. Each of these BEI controls was subjected to library preparation and sequencing twice as technical replicates.

All species present in the mock community controls were identified. The error rate for the Zymo mock community control was 0.005% when the data was processed with the *dada2* pipeline.^3^ For the BEI mock community controls, the error rate was 0.006% when the data was processed with the *dada2* pipeline.

***16S ribosomal RNA sequencing data processing and statistical analyses***

Both pediatric and adult sequencing reads were processed concurrently with the R^4^ package *dada2*^3^ standard operating procedure in order to generate a single amplicon sequence variant (ASV) table, which was used for all downstream analyses. Taxonomy was assigned with the Ribosomal Database Project database.^5^ Sequences were subsequently processed through the R^4^ package *decontam* with the “prevalence” method to remove suspected contaminants that had been identified in the negative control samples.^6^ The R *phyloseq* package was used to facilitate data processing.^7^

The statistical analyses were conducted in R,^4^ mostly using the open-source package MGSAT,^8^ as previously described.^9-12^ The MGSAT pipeline wraps several R packages to compare common microbial ecology indices of community richness, α-diversity, and structure, as well as the abundance of taxa, between groups. It applies several types of statistical tests, data normalizations, and plotting routines to the abundance count matrices that are typically the output of annotating (meta) omics datasets, generating a structured HTML report that shows the results, method parameters, and versions of the external packages. The user has fine-grained control over the statistical tests, parameters, and a description of a study design through a data structure that is provided as input to the top-level routine of the package.

To compare the community richness and α-diversity between groups, we used the R *vegan* package,^13^ as implemented in MGSAT. All ASVs were included in estimates of community richness and α-diversity, regardless of abundance. To control for differences in sequencing depths, counts were randomly rarefied to the lowest library size of all samples (i.e., n=1,366) and then common microbial ecology indices were computed (e.g., Chao1, Jackknife, Bootstrap, Shannon, and Simpson). For each index, this rarefaction and computation process was repeated multiple times (n=400) and the results were averaged. Linear models were then fit to test for associations between community richness or α-diversity estimates and sample type.

To compare the community structure between groups, we also used the R *vegan* package,^13^ as implemented in MGSAT. For this, we used the Bray-Curtis dissimilarity index computed on simple proportions (on which it is equivalent to the Manhattan index) and 4,000 permutations.

The PermANOVA (*Adonis* function) and *betadisper* tests were then used to test for differences in Bray-Curtis dissimilarities and homogeneity of variances between groups, respectively.^14^ For the PermANOVA tests, strata was set as subject study numbers. The same rarefaction process described in the previous paragraph was applied.

To compare the abundance of taxa between groups, we used the *DESeq2* test,^15^ as implemented in MGSAT. For this, we conducted unbiased metadata-independent filtering at each taxonomy level by eliminating all taxa that were detected on average <10 times, taxa with a minimum quantile mean fraction <0.1, and taxa with a minimum quantile incidence fraction <0.1. Following this step, 75 genera and 157 ASVs remained for testing. The absolute counts from the removed features were aggregated into a category “other”, which was taken into account when computing simple proportions during data normalization for subsequent plotting but were otherwise discarded. This was done to reduce the penalty associated to multiple comparisons and remove likely non-informative data. *DESeq2* testing was run by pooling pediatric and adult samples together in order to use the same taxa count data table for both comparisons. Contrasts within *DESeq2* were used to examine the log2-fold changes and *q*-values for 1) pediatric nasal filters compared to pediatric nasal washes, and 2) adult nasal filters compared to adult nasal washes. The *DESeq2* test uses shrinkage estimation for dispersions and fold changes to improve the stability and interpretability of estimates. This method models raw absolute counts of each taxon with a negative binomial distribution and uses the estimated depth of sequencing of each sample to scale the (unknown) relative abundance that is the parameter of the negative binomial distribution. Compared to using either simple proportion-based normalization or rarefaction to control for differences in sequencing depth, the *DESeq2* test provides improved sensitivity and specificity.^16^ We included subject study number as a covariate in all *DESeq2* models. Reported *q*-values are the result of a Wald test with the Benjamini-Hochberg correction for multiple comparisons.^17^ Default outlier detection and replacement was used as described in the original *DESeq2* publication.^15^

Because the number of paired samples with shotgun metagenomic sequencing data was small, we did not perform any statistical comparisons between groups and only compared these using descriptive statistics.

The statistical analyses of the immune mediator dataset were also conducted in R.^4^ The median fluorescence intensity (MFI) of each of the 53 analytes was calculated after subtracting out the background MFI and this was used for all statistical analyses. Because the MFIs were not normally distributed, these were log10 transformed. Prior to this, all negative MFIs were set to 0, as a value <0 indicates that the MFI for that particular analyte was lower than the background MFI (i.e., that the analyte was not truly detected in that sample). The MFIs were then transformed using the equation log10(x+1) to allow analyte readings of 0 to remain 0. The comparisons of MFIs between paired samples were performed using a Wilcoxon signed-rank test with the Benjamini-Hochberg correction to control for multiple comparisons.^17^

For data visualization, we used grouped or stacked bar graphs, box and whisker plots, heatmaps, and ordination plots based on different microbial ecology indices or immune mediators MFIs, as appropriate. Figures were generated with the R packages *ggplot2*,^18^ *vegan*,^19^ or *ComplexHeatmap*,^20^ as appropriate. Heatmaps were generated with *ComplexHeatmap*;^20^ the Pearson correlation was used to calculate dissimilarities. The number of cluster splits was determined by partitioning around medoids (method pamk in R package *fpc* ^21^). Minor aesthetic edits to the figures were made with Inkscape, version 1.0. Statistical significance was defined as *p*- or *q*-values <0.05. Due to the differences in sample processing steps between children and adults, all results are presented separately for each of these age groups.

| **Table E1.** Upper airway immune mediators tested in adult paired samples as part of this study. | | |
| --- | --- | --- |
| - BCA-1 - CTACK - EGF - ENA-78 - Eotaxin - Eotaxin-2 - Eotaxin-3 - FGF-basic - G-CSF - GM-CSF - HGF - IFN-α - IFN-γ - IL-1β - IL-1RA - IL-2 - IL-2R - IL-4 - IL-5 - IL-6 | - IL-7 - IL-8 - IL-10 - IL-12 - IL-13 - IL-15 - IL-16 - IL-17A - IL-20 - IL-21 - IL-23 - IL-28A - IL-33 - IP-10 - I-309 - LIF - MCP-1 - MCP-2 - MCP-4 - MIG | - MIP-1α - MIP-1β - MIP-1d - RANTES - SCF - SDF-1A+β - TARC - TNF-α - TPO - TRAIL - TSLP - VEGF - 6Ckine |

**Table E2.** Upper airway genera abundance in children and adults with paired samples based on 16S ribosomal RNA sequencing and according to sample type.^*†^

| *Genus* | *Children* | | *Adults* | |  |
| --- | --- | --- | --- | --- | --- |
|  | *Log2-fold change*^‡^ | *q-value*^§^ | *Log2-fold change*^‡^ | *q-value*^§^ |  |
| *Abiotrophia* | -3.6419 | 1 | 14.0431 | 0.0008 |  |
| *Actinomyces* | -1.7218 | 0.9754 | -7.7940 | 7.90E-07 |  |
| *Alloprevotella* | -1.9149 | 1 | -24.9149 | 4.23E-29 |  |
| *Anaerococcus* | 0.0384 | 1 | -7.5883 | 0.0359 |  |
| *Atopobium* | -1.2858 | 1 | -26.1687 | 1.85E-52 |  |
| *Bacteria_*unclassified | -4.6224 | 0.0889 | -4.2120 | 0.0359 |  |
| *Camplylobacter* | -0.4588 | 1 | -4.8629 | 0.0068 |  |
| *Capnocytophaga* | -3.0843 | 0.6507 | -10.5020 | 4.56E-07 |  |
| *Corynebacterium* | -0.9393 | 1 | -5.2586 | 0.0002 |  |
| *Dialister* | 7.0607 | 0.6507 | -17.3490 | 0.0004 |  |
| *Finegoldia* | 0.0000 | 1 | -14.0360 | 0.0052 |  |
| *Fusobacterium* | -0.0356 | 1 | -29.3775 | 1.97E-42 |  |
| *Gemella* | -1.7631 | 1 | -5.4149 | 0.0083 |  |
| *Granulicatella* | -1.0307 | 1 | -29.8131 | 7.91E-57 |  |
| *Haemophilus* | -0.2757 | 1 | -9.2354 | 1.56E-10 |  |
| *Leptotrichia* | -1.4512 | 1 | -18.2994 | 2.15E-20 |  |
| *Megasphaera* | -1.4223 | 1 | -25.6842 | 3.06E-32 |  |
| *Moraxella* | 3.0553 | 0.8146 | -6.4130 | 0.0126 |  |
| *Neisseria* | -1.2884 | 1 | -29.9851 | 6.15E-62 |  |
| *Porphyromonas* | -3.2087 | 0.4264 | -29.3007 | 2.27E-56 |  |
| *Prevotella* | -1.7559 | 0.9754 | -7.4536 | 9.99E-06 |  |
| *Prevotellaceae_*unclassified | 6.6605 | 0.6507 | -28.3225 | 1.47E-09 |  |
| *Propionibacterium* | 0.1072 | 1 | -5.0489 | 0.0083 |  |
| *Rothia* | -3.1327 | 0.4264 | -11.3913 | 4.30E-10 |  |
| *Selenomonas* | -1.1675 | 1 | -20.6853 | 1.81E-19 |  |
| *Sphingobium* | -24.4456 | 3.93E-16 | 6.7615 | 0.0711 |  |
| *SR1_*unclassified | 2.3141 | 1 | -16.5245 | 0.0008 |  |
| *Staphylococcus* | -1.9853 | 0.9754 | -6.6965 | 0.0001 |  |
| *Streptococcus* | -1.4655 | 0.6507 | -3.7183 | 0.0004 |  |
| *Veillonella* | -1.7028 | 0.9754 | -4.5044 | 0.0131 |  |
| ^*^ Data presented as results of the *DESeq2* test. The *DESeq2* models included subject ID as a covariate.  ^†^Only genera that were different between sample types in the *DESeq2* test (*q*-value<0.05) for either children or adults are shown.  ^‡^A negative log2-fold change indicates that the particular genera was more abundant in nasal filters than in nasal washes, whereas a positive log2-fold change indicates the opposite.  ^§^*q*-values are the result of a Wald test with the Benjamini-Hochberg correction to control for multiple comparisons. | | | | | |

**Table E3.** Upper airway ASV abundance in children and adults with paired samples based on 16S ribosomal RNA sequencing and according to sample type.^*†^

| *ASV* | *Children* | | *Adults* | |
| --- | --- | --- | --- | --- |
|  | *Log2-fold change*^‡^ | *q-value*^§^ | *Log2-fold change*^‡^ | *q-value*^§^ |
| *Acidovorax*_unclassified.ASV0065 | 1.2272 | 0.9982 | 14.4053 | 0.0029 |
| *Actinomyces*_*odontolyticus*.ASV0023 | -1.4106 | 0.9982 | -14.0126 | 1.15E-07 |
| *Actinomyces*_*odontolyticus*.ASV0099 | -11.8630 | 0.0195 | -24.6828 | 2.51E-07 |
| *Actinomyces*_unclassified.ASV0124 | 4.4743 | 0.8429 | -21.6017 | 6.41E-06 |
| *Alloprevotella*_unclassified.ASV0057 | -5.9249 | 0.2791 | -14.0442 | 0.0002 |
| *Anaerococcus*_unclassified.ASV0138 | -1.9110 | 0.9982 | -12.1845 | 0.0147 |
| *Candidatus*_*Saccharibacteria*_unclassified.ASV0051 | -6.8565 | 0.3729 | -17.2762 | 0.0003 |
| *Candidatus*_*Saccharibacteria*_unclassified.ASV0144 | -8.9596 | 0.1259 | -20.3400 | 2.16E-05 |
| *Capnocytophaga*_*gingivalis*.ASV0153 | 11.0550 | 0.0337 | -21.3247 | 8.47E-06 |
| *Capnocytophaga*_*sputigena*.ASV0076 | -10.0651 | 0.0514 | -14.6322 | 0.0022 |
| *Clostridiales*_unclassified.ASV0059 | -10.5130 | 0.0477 | -29.3942 | 1.26E-09 |
| *Corynebacterium*_*kroppenstedtii*.ASV0093 | -1.7442 | 0.9982 | -14.5305 | 0.0029 |
| *Corynebacterium*_unclassified.ASV0031 | -0.1710 | 1 | -12.2721 | 0.0142 |
| *Corynebacterium*_unclassified.ASV0067 | -7.2426 | 0.3201 | -18.7277 | 9.33E-05 |
| *Dolosigranulum*_*pigrum*.ASV0036 | -2.1118 | 0.9982 | -17.3387 | 0.0003 |
| *Erysipelothrix*_unclassified.ASV0103 | -1.3678 | 0.9982 | 11.2934 | 0.0252 |
| *Flavobacteriaceae*_unclassified.ASV0083 | -3.8456 | 0.9982 | -18.4371 | 0.0001 |
| *Fusobacterium*_unclassified.ASV0071 | -5.8607 | 0.4765 | -18.2583 | 4.27E-05 |
| *Fusobacterium*_unclassified.ASV0164 | -12.2974 | 0.0168 | -18.4875 | 0.0001 |
| *Gemella*_unclassified.ASV0052 | -2.1686 | 0.9982 | -13.0047 | 3.51E-06 |
| *Granulicatella*_*elegans*.ASV0077 | -2.9113 | 0.9982 | -19.7376 | 3.76E-05 |
| *Granulicatella*_unclassified.ASV0043 | -2.5394 | 0.7916 | -16.5968 | 2.06E-09 |
| *Haemophilus*_*parainfluenzae*.ASV0004 | -2.2169 | 0.7916 | -12.9753 | 1.23E-07 |
| *Haemophilus*_unclassified.ASV0049 | 24.6958 | 9.08E-09 | -1.9342 | 0.7564 |
| *Haemophilus*_unclassified.ASV0050 | 0.4259 | 0.9982 | -16.9505 | 1.22E-05 |
| *Lautropia*_*mirabilis*.ASV0081 | -4.4976 | 0.6841 | -18.4089 | 4.58E-06 |
| *Leptotrichia*_unclassified.ASV0115 | -12.7407 | 0.0122 | -29.5507 | 1.26E-09 |
| *Megasphaera*_*micronuciformis*.ASV0024 | -1.3249 | 0.9982 | -18.0634 | 1.26E-09 |
| *Moraxella*_unclassified.ASV0007 | 7.5119 | 0.2891 | -18.1473 | 0.0002 |
| *Neisseria*_unclassified.ASV0011 | -1.0406 | 0.9982 | -14.1030 | 1.42E-06 |
| *Neisseria*_unclassified.ASV0022 | -2.9508 | 0.7916 | -11.4372 | 0.0002 |
| *Neisseria*_unclassified.ASV0104 | -11.8132 | 0.0195 | -29.7985 | 1.26E-09 |
| *Opitutus*_unclassified.ASV0111 | -0.7396 | 0.9982 | 9.5493 | 0.0483 |
| *Oribacterium*_*asaccharolyticum*.ASV0113 | -5.5652 | 0.6581 | -29.5047 | 1.26E-09 |
| *Porphyromonas*_unclassified.ASV0020 | -2.7868 | 0.7916 | -15.1730 | 1.93E-07 |
| *Prevotella*_*histicola*.ASV0005 | -2.6875 | 0.7916 | -14.2717 | 2.13E-07 |
| *Prevotella*_*melaninogenica*.ASV0002 | -2.8005 | 0.4889 | -11.0847 | 2.16E-06 |
| *Prevotella*_*melaninogenica*.ASV0097 | -1.9425 | 0.9982 | -26.4629 | 4.32E-08 |
| *Prevotella*_*nanceiensis*.ASV0058 | -3.4693 | 0.9982 | -26.9744 | 2.53E-08 |
| *Prevotella*_*oris*.ASV0088 | -12.0532 | 0.0188 | -23.7932 | 6.68E-07 |
| *Prevotella*_*oulorum*.ASV0100 | 11.6335 | 0.0223 | -25.0508 | 1.93E-07 |
| *Prevotella*_*pallens*.ASV0013 | -3.4670 | 0.7916 | -19.1349 | 4.32E-08 |
| *Prevotella*_*pallens*.ASV0029 | -14.6855 | 0.0030 | -23.9715 | 5.59E-07 |
| *Prevotella*_*salivae*.ASV0012 | -1.6864 | 0.9982 | -11.0194 | 3.45E-05 |
| *Prevotella*_*shahii*.ASV0162 | -12.0272 | 0.0188 | -20.8747 | 1.31E-05 |
| *Prevotella*_unclassified.ASV0044 | -0.5997 | 0.9982 | -19.7076 | 3.79E-05 |
| *Prevotella*_unclassified.ASV0072 | 14.5969 | 0.0032 | -23.0735 | 1.42E-06 |
| *Prevotella*_unclassified.ASV0074 | 15.6200 | 0.0016 | -11.5080 | 0.0242 |
| *Prevotella*_unclassified.ASV0101 | -0.8992 | 0.9982 | -26.9645 | 2.53E-08 |
| *Prevotella*_unclassified.ASV0123 | -6.3927 | 0.4765 | -25.7491 | 1.01E-07 |
| *Prevotella*_unclassified.ASV0128 | -34.1942 | 1.67E-16 | -13.5913 | 0.0061 |
| *Prevotella*_unclassified.ASV0142 | -34.5114 | 1.62E-16 | -13.5305 | 0.0063 |
| *Prevotella*_unclassified.ASV0148 | -10.3626 | 0.0514 | -20.6826 | 1.57E-05 |
| *Prevotella*_unclassified.ASV0171 | 29.9101 | 1.12E-12 | -9.5648 | 0.0722 |
| *Prevotella*_*veroralis*.ASV0127 | -1.2229 | 0.9982 | -24.3147 | 3.81E-07 |
| *Rothia*_*mucilaginosa*.ASV0122 | -9.8743 | 0.0698 | -19.5128 | 4.43E-05 |
| *Selenomonas*_*infelix*.ASV0098 | 15.0519 | 0.0025 | -8.3570 | 0.1239 |
| *Selenomonas*_unclassified.ASV0105 | -12.9069 | 0.0114 | -20.1812 | 2.49E-05 |
| *SR1*_unclassified.ASV0056 | -13.9754 | 0.0045 | -10.9474 | 0.0336 |
| *SR1*_unclassified.ASV0133 | -1.1779 | 0.9982 | -22.7884 | 1.86E-06 |
| *Staphylococcus*_unclassified.ASV0006 | -0.8177 | 0.9982 | -5.6099 | 0.0028 |
| *Streptococcus*_*sanguinis*.ASV0129 | -14.2772 | 0.0038 | -23.1686 | 1.32E-06 |
| *Streptococcus*_unclassified.ASV0010 | -2.5583 | 0.7916 | -9.4220 | 0.0004 |
| *Streptococcus*_unclassified.ASV0019 | 7.4171 | 0.2942 | -24.8839 | 2.13E-07 |
| *Streptococcus*_unclassified.ASV0034 | -5.0194 | 0.7916 | -23.5647 | 8.48E-07 |
| *Streptococcus*_unclassified.ASV0135 | -14.2071 | 0.0039 | -17.3187 | 0.0003 |
| *Streptococcus*_unclassified.ASV0151 | 0.0000 | 1 | -10.7017 | 0.0354 |
| *Veillonella*_unclassified.ASV0001 | -1.6094 | 0.9982 | -12.2494 | 2.09E-07 |
| *Veillonella*_unclassified.ASV0016 | -1.1514 | 0.9982 | -15.7205 | 1.26E-09 |
| *Veillonella*_unclassified.ASV0030 | -0.9205 | 0.9982 | -27.2137 | 2.24E-08 |
| *Veillonella*_unclassified.ASV0032 | -5.2881 | 0.2808 | -14.4997 | 2.16E-05 |
| *Veillonella*_unclassified.ASV0062 | -1.7955 | 0.9982 | -19.0540 | 6.87E-05 |
| *Veillonella*_unclassified.ASV0125 | -24.7127 | 9.08E-09 | -6.3926 | 0.2820 |
| *Definition of abbreviations:* ASV = Amplicon sequence variant.  ^*^Data presented as results of the *DeSeq2* test. The *DeSeq2* models included subject ID as a covariate.  ^†^Only ASVs that were different between sample types in the *DeSeq2* test (*q*-value<0.05) for either children or adults are shown.  ^‡^A negative log2-fold change indicates that the particular ASV was more abundant in nasal filters than in nasal washes, whereas a positive log2-fold change indicates the opposite.  ^§^*q*-values are the result of a Wald test with the Benjamini-Hochberg correction to control for multiple comparisons. | | | | |

| **Table E4.** Upper airway immune mediators in adults with paired samples according to sample type.^*^ | | | |
| --- | --- | --- | --- |
| *Immune mediator* | *Nasal filters*^†^ | *Nasal washes*^†^ | *q-value*^‡^ |
| BCA-1 | 1.6732 (0.7876-2.0139) | 0 (0-0.1321) | 0.0057505 |
| CTACK | 0.7404 (0.3618-0.9083) | 0 (0-0) | 0.02494614 |
| EGF | 1.8862 (1.7191-1.9554) | 0 (0-0) | 0.0057505 |
| ENA-78 | 1.7011 (0.8415-1.9466) | 0 (0-0) | 0.01154895 |
| Eotaxin | 1.2665 (1.0595-1.3374) | 0 (0-0) | 0.0057505 |
| Eotaxin-2 | 2.4939 (2.2671-2.7309) | 0 (0-0.3578) | 0.01154895 |
| Eotaxin-3 | 0.3979 (0.301-0.6259) | 0 (0-0) | 0.0057505 |
| FGF-basic | 1.4065 (1.3253-1.4784) | 0 (0-0) | 0.008003 |
| G-CSF | 1.3972 (1.2955-1.4546) | 0 (0-0.1321) | 0.0057505 |
| GM-CSF | 0.699 (0.5714-0.7404) | 0 (0-0) | 0.008003 |
| HGF | 1.4393 (1.1686-1.5523) | 0 (0-0) | 0.0057505 |
| IFN-α | 1.2364 (1.0966-1.3169) | 0 (0-0) | 0.008003 |
| IFN-γ | 1.2269 (1.1513-1.2492) | 0.2430 (0.1334-0.243) | 0.0057505 |
| IL-1β | 1.1758 (1.0636-1.3638) | 0 (0-0) | 0.0057505 |
| IL-1RA | 3.906 (3.8987-3.9113) | 0.6901 (0-1.2579) | 0.0057505 |
| IL-2 | 1.317 (1.2256-1.3683) | 0 (0-0) | 0.0057505 |
| IL-2R | 1.1054 (0.9985-1.1461) | 0.1761 (0.1761-0.2698) | 0.008003 |
| IL-4 | 1.29 (1.1821-1.3222) | 0 (0-0) | 0.008003 |
| IL-5 | 0.6021 (0.5084-0.6404) | 0 (0-0) | 0.008003 |
| IL-6 | 1.636 (1.5553-1.744) | 0 (0-0) | 0.0057505 |
| IL-7 | 0.7955 (0.6366-0.8371) | 0 (0-0) | 0.008003 |
| IL-8 | 3.7824 (3.227-3.8415) | 1.525 (1.4008-1.5425) | 0.0057505 |
| IL-10 | 0.6761 (0.5441-0.7687) | 0 (0-0.1761) | 0.008003 |
| IL-12 | 1.1968 (1.0403-1.2627) | 0 (0-0) | 0.008003 |
| IL-13 | 0.7404 (0.5587-0.7782) | 0 (0-0) | 0.008003 |
| IL-15 | 1.4065 (1.3148-1.4637) | 0 (0-0) | 0.0057505 |
| IL-16 | 2.556 (1.4068-2.9788) | 0 (0-0) | 0.0057505 |
| IL-17A | 1.2173 (0.9983-1.2522) | 0.1761 (0.044-0.1761) | 0.008003 |
| IL-20 | 0.2386 (0.044-0.4573) | 0 (0-0) | 0.02591239 |
| IL-21 | 0.1761 (0-0.2698) | 0 (0-0) | 0.06325388 |
| IL-23 | 0.3495 (0.044-0.5708) | 0 (0-0) | 0.02591239 |
| IL-28A | 0 (0-0) | 0 (0-0) | 0.3711 |
| IL-33 | 0.7766 (0.0995-1.0207) | 0 (0-0) | 0.03357043 |
| IP-10 | 1.4787 (1.4334-1.5899) | 0.301 (0.301-0.301) | 0.0057505 |
| I-309 | 0.5106 (0.2316-0.6259) | 0 (0-0) | 0.008003 |
| LIF | 0 (0-0.2698) | 0 (0-0) | 0.10423333 |
| MCP-1 | 2.2273 (1.8525-2.2642) | 0.2386 (0.1761-0.3737) | 0.0057505 |
| MCP-2 | 0.5106 (0.4177-0.5876) | 0 (0-0) | 0.01154895 |
| MCP-4 | 0.5441 (0.4939-0.5876) | 0 (0-0) | 0.008003 |
| MIG | 0.9404 (0.8024-1.0671) | 0 (0-0) | 0.008003 |
| MIP-1α | 1.1054 (0.9855-1.1381) | 0 (0-0) | 0.008003 |
| MIP-1β | 1.1136 (1.0198-1.1576) | 0 (0-0) | 0.008003 |
| MIP-1d | 1.6084 (0.7219-2.1689) | 0 (0-0) | 0.008003 |
| RANTES | 0.7386 (0.6263-0.8371) | 0 (0-0) | 0.008003 |
| SCF | 0.6532 (0.3451-0.8086) | 0 (0-0) | 0.008003 |
| SDF-1A+β | 0.3495 (0.044-0.3979) | 0 (0-0) | 0.05502063 |
| TARC | 1.0299 (0.5828-1.2801) | 0 (0-0.1761) | 0.008003 |
| TNF-α | 0.9889 (0.9007-1.0647) | 0 (0-0) | 0.008003 |
| TPO | 0 (0-0.4081) | 0 (0-0) | 0.18488846 |
| TRAIL | 3.4923 (2.7499-3.7595) | 0 (0-0.1321) | 0.0057505 |
| TSLP | 0.6021 (0.2316-0.6532) | 0 (0-0) | 0.01846372 |
| VEGF | 2.4862 (2.4643-2.5072) | 0.2386 (0-0.3737) | 0.0057505 |
| 6Ckine | 0 (0-0.2698) | 0 (0-0) | 0.10423333 |
| ^*^The immune mediators are presented in alphabetical order.  ^†^Data presented as median (interquartile range).  ^‡^*q*-values are the result of a Wilcoxon signed-rank test with the Benjamini-Hochberg correction to control for multiple comparisons. A shaded cell indicates a *q*-value<0.05. | | | |

**Supplementary Figure Legends**

**Supplementary Figure 1.** Venn diagrams showing the distribution of shared and unique amplicon sequence variants (ASVs) between nasal filters and washes are presented for **A)** pediatric subjects and **B)** adult subjects. Venn diagrams were generated with the online tool Venn Diagram Maker Online (<https://www.meta-chart.com/venn#/>).

**Supplementary Figure 2.** Rarefaction curves are shown for whole metagenomic shotgun sequencing data. Richness (number of observed bacterial species) was calculated at different sampling depths. For 9 of the 12 samples, rarefaction curves level off, indicating that sequencing was performed to a sufficient depth for the majority of the samples. This figure was generated with the R^4^ package *ggplot2* version 3.0.0 (https://cran.r-project.org/web/packages/ggplot2/index.html).^18^

**E-References**

1 Kozich, J. J., Westcott, S. L., Baxter, N. T., Highlander, S. K. & Schloss, P. D. Development of a dual-index sequencing strategy and curation pipeline for analyzing amplicon sequence data on the MiSeq Illumina sequencing platform. *Applied and environmental microbiology* **79**, 5112-5120, doi:10.1128/aem.01043-13 (2013).

2 Baker, R. & Peacock, S. BEI Resources: supporting antiviral research. *Antiviral research* **80**, 102-106, doi:10.1016/j.antiviral.2008.07.003 (2008).

3 Callahan, B. J. *et al.* DADA2: High-resolution sample inference from Illumina amplicon data. *Nat Methods* **13**, 581-583, doi:10.1038/nmeth.3869 (2016).

4 R Core Team. R: A Language and Environment for Statistical Computing (Vienna, Austria, 2019).

5 Cole, J. R. *et al.* The Ribosomal Database Project: improved alignments and new tools for rRNA analysis. *Nucleic acids research* **37**, D141-145, doi:10.1093/nar/gkn879 (2009).

6 Davis, N. M., Proctor, D. M., Holmes, S. P., Relman, D. A. & Callahan, B. J. Simple statistical identification and removal of contaminant sequences in marker-gene and metagenomics data. *Microbiome* **6**, 226, doi:10.1186/s40168-018-0605-2 (2018).

7 McMurdie, P. J. & Holmes, S. phyloseq: an R package for reproducible interactive analysis and graphics of microbiome census data. *PloS one* **8**, e61217, doi:10.1371/journal.pone.0061217 (2013).

8 Tovchigrechko, A. *MGSAT - Statistical analysis of microbiome and proteome abundance matrices with automated report generation*, <<https://github.com/andreyto/mgsat>> (2015).

9 Rosas-Salazar, C. *et al.* Differences in the Nasopharyngeal Microbiome During Acute Respiratory Tract Infection With Human Rhinovirus and Respiratory Syncytial Virus in Infancy. *The Journal of infectious diseases* **214**, 1924-1928, doi:10.1093/infdis/jiw456 (2016).

10 Shilts, M. H. *et al.* Minimally Invasive Sampling Method Identifies Differences in Taxonomic Richness of Nasal Microbiomes in Young Infants Associated with Mode of Delivery. *Microb Ecol* **71**, 233-242, doi:10.1007/s00248-015-0663-y (2016).

11 Rosas-Salazar, C. *et al.* Nasopharyngeal Lactobacillus is Associated with Childhood Wheezing Illnesses Following Respiratory Syncytial Virus Infection in Infancy. *J. Allergy Clin. Immunol.* **142**, 1447-1456 (2018).

12 Rosas-Salazar, C. *et al.* Nasopharyngeal Microbiome in Respiratory Syncytial Virus Resembles Profile Associated with Increased Childhood Asthma Risk. *Am. J. Respir. Crit. Care Med.* **193**, 1180-1183, doi:10.1164/rccm.201512-2350LE (2016).

13 Oksanen, J. *et al.* *vegan: Community Ecology Package*, <<http://CRAN.R-project.org/package=vegan>> (2014).

14 Anderson, M. J. A new method for non-parametric multivariate analysis of variance. *Austral Ecology* **26**, 32-46, doi:DOI 10.1111/j.1442-9993.2001.01070.pp.x (2001).

15 Love, M. I., Huber, W. & Anders, S. Moderated estimation of fold change and dispersion for RNA-seq data with DESeq2. *Genome Biol* **15**, 550, doi:10.1186/s13059-014-0550-8 (2014).

16 McMurdie, P. J. & Holmes, S. Waste not, want not: why rarefying microbiome data is inadmissible. *PLoS computational biology* **10**, e1003531, doi:10.1371/journal.pcbi.1003531 (2014).

17 Benjamini, Y. & Hochberg, Y. Controlling the False Discovery Rate - a Practical and Powerful Approach to Multiple Testing. *Journal of the Royal Statistical Society Series B-Methodological* **57**, 289-300 (1995).

18 Wickham, H. *ggplot2: elegant graphics for data analysis*. (Springer, 2009).

19 Oksanen, J. *et al.* vegan: Community Ecology Package. R package version 2.0-10. (2014).

20 Gu, Z., Eils, R. & Schlesner, M. Complex heatmaps reveal patterns and correlations in multidimensional genomic data. *Bioinformatics* **32**, 2847-2849, doi:10.1093/bioinformatics/btw313 (2016).

21 Hennig, C. *Flexible Procedures for Clustering. R package version 2.1-11.1*, <<https://CRAN.R-project.org/package=fpc>> (2018).
